# Prevalence of problem drinking in the Swedish workforce: differences between labour market industries based on gender composition and main job activity

**DOI:** 10.1186/s12889-024-20163-y

**Published:** 2024-10-01

**Authors:** Hasan Tareq, Anna Nyberg, Peter Wennberg, David Redmalm, Susanna Toivanen, Aziz Mensah

**Affiliations:** 1 School of Health, Care, and Social Welfare, Mälardalen University, Universitetsplan 1, Västerås, 722 20 Sweden; 2 Department of Public Health and Caring Sciences, Uppsala University, Uppsala, Sweden; 3 Department of Public Health Sciences, Stockholm University, Stockholm, Sweden; 4 Department of Psychology, Inland Norway University of Applied Sciences, Lillehammer, Norway; 5 Unit of Intervention and Implementation Research for Worker Health, Institute for Environmental Medicine, Karolinska Institutet, Stockholm, Sweden

**Keywords:** Problem drinking, Alcohol, Prevalence, Labour Market, Occupational Health, Gender differences, Gender-typed industries, Public Health, Cross-sectional study

## Abstract

**Background:**

Identifying problem drinking patterns across industries is essential for addressing drinking problems in the workforce. Still, it is not well understood how problem drinking differs across industries and whether it is associated with industry gender composition. This study aimed to measure the prevalence of problem drinking (PPD) across Swedish industries and investigate possible associations between gender-typed industries and problem drinking.

**Methods:**

9,155 current workers were selected from the Swedish Longitudinal Occupational Survey of Health (SLOSH) data collected in 2020. Participants’ work industries were identified through the Swedish Standard Industrial Classification (SNI) codes. Seven gender-typed industry categories were created based on gender composition and main job activity in each industry. Self-reported problem drinking was measured using a slightly modified Cut-down, Annoyed, Guilt, Eye-opener (CAGE) questionnaire and a cut-off score 2 was used to determine problem drinking. Poisson regression with robust standard errors was used to investigate the association between gender-typed industries and problem drinking.

**Results:**

PPD in the workforce was 6.6%. Men (8.5%) had a higher prevalence than women (5.3%). Across industries, PPD varied from 2.3% in Water supply and waste management to 15.4% in Mining and quarrying. The highest prevalence for men was in Mining and quarrying (18.2%), whereas for women it was in Construction (11.1%). Within gender-typed industries, the highest PPD was in male-dominated Goods and Energy Production (7.7%), and the lowest was in female-dominated Health and Social Care (4.7%). In the regression analysis, both Education (aPR: 1.39, *p* = 0.03) and Labour-intensive Services (aPR: 1.39, *p* = 0.02) had higher adjusted prevalence ratios (aPR) compared with Health and Social Care. However, there was no significant difference in aPR among gender-typed industries when considering the gender composition of industries only.

**Conclusions:**

PPD in the Swedish workforce varied significantly across industries, with differences observed between men and women. Problem drinking differed between industries when categorized by gender composition and main job activity, but not when categorized by gender composition only. Future research should investigate how industry-specific psychosocial factors influence individual alcohol consumption.

**Supplementary Information:**

The online version contains supplementary material available at 10.1186/s12889-024-20163-y.

## Introduction

Problem drinking in the working population not only affects employee health but also the work environment and industry productivity. Previous research has shown that problem drinking is associated with decreased productivity [1], increased absenteeism [2], a higher incidence of workplace injuries [3], and negative effects on the work environment [3–5]. To address this concern, it is important to understand problem drinking in the working population from a broader perspective.

Previous studies have shown that male workers, on average, consume more alcohol than their female counterparts [6–10]. However, the drinking patterns of men and women across industries and whether the gender composition of industries affects working population’s drinking patterns are not well understood.

Despite similar labour force participation rates in women and men in Sweden, the labour market is structured by gender, resulting in varying compositions of male and female workers in different industries and professional groups [11]. Gender differences in the labour market play a critical role in shaping the overall work environment and might potentially influence employees’ alcohol consumption [12–17]. Various studies from the United States [17, 18], Finland [19], and Sweden [20] have indicated positive associations between women working in male-dominated occupations and increased drinking. In contrast, another study from the US investigated women workers’ drinking based on both gender composition of industry and occupation and reported that women are more likely to drink more in gender-mixed industries than in male-dominated industries [21]. On the other hand, other studies reach different conclusions. For example, a study from Sweden did not find any association between male-dominated occupations and problem drinking in women in those occupations [22]. This study, however, had a small sample size restricted to a particular geographic region in Sweden, limiting its generalisability.

A major limitation of existing research on this topic is the predominant focus on occupational gender composition, which generally categorises jobs based on proportion of men and women within specific occupations. However, occupational categories may not fully capture the gender dynamics of work environments, work cultures and associated stressors which can vary significantly between industries such as health care, manufacturing, education etc. Occupational health researchers Härenstam and Nyberg compared existing approaches to investigating gender differences in the labour market and their association with work-related health outcomes and suggested an alternative approach to exploring this complex relationship [13]. They argued that because work objectives, tasks and production technologies are gendered, a structural rather than individual perspective should be applied. Technology-intensive production is, for example, traditionally linked to masculinity, both in terms of its products and services and in terms of the bodies performing the work tasks. Meeting the needs of other human beings is, on the other hand, traditionally connected to femininity in both of these respects. Due to different organisational prerequisites between industries with different job activities and gender domination, there are systematic differences in the work environment and health risks for men and women in the labour market. To address this in survey and register-based research, Härenstam and Nyberg suggested an alternative approach of industry categorization by considering both work type and gender composition of an industry [13]. For example, both the health and social care industry and the educational industry are female-dominated, but the main work objectives differ between these industries and therefore they are separated in their categorization. The same applies to male-dominated and gender-integrated industries. Another study used a similar industry categorization to explore changes in psycho-social work environment in Swedish gender-typed industries between 1992 and 2013 [23]. The study suggested that future research should follow this approach, combining both gender composition and industry job activity, as the gender composition of industries also changes with shifts in job activity and technological adaptation.

Furthermore, while some existing studies have primarily focused on women workers’ drinking patterns within gender-typed workforces [17, 19, 21, 22], the gender composition of the labour market may also influence men’s drinking behaviours, either positively or negatively, which is also worth exploring.

To the best of our knowledge, no study has systematically measured the industry-specific prevalence of problem drinking or investigated possible associations between gender-typed industries and problem drinking in the Swedish workforce. The objective of this study is to map the prevalence of problem drinking in the Swedish workforce and investigate possible associations between gender-typed industries and problem drinking in employees.

## Method

### Study population

We used data from the Swedish Longitudinal Occupational Survey of Health (SLOSH), which is a cohort study based on repeated follow-ups of the Swedish Work Environment Survey (SWES). SLOSH has been collecting work and health-related data from Swedish working individuals since 2006, regardless of their current employment status [24]. More detailed information about the SLOSH study is available at www.slosh.se.

For this study, we identified 9,712 participants from the SLOSH 2020 survey respondents who were employed and working at least 30% of full-time hours or 48 h per month. Additional demographic information, such as age, education, marital status, and work industry was obtained from population registries managed by Statistics Sweden and linked to the SLOSH questionnaire data. After excluding individuals with missing or incomplete data in key variables like work industry (1.4%), education (0.1%), civil status (1.4%), and alcohol consumption behaviour (1.4%), the final sample consisted of 9,155 individuals, including 5,298 women (57.9%) and 3,857 men (42.1%).

### Measures

#### Gender-typed industries

The work industry of each individual was identified using the 2007 version of the Swedish Standard Industrial Classification (SNI), developed based on recommendations of the statistical classification of economic activities in the European Community (NACE) [25]. SNI 2007 contains 21 major industry categories, 19 of which are associated with paid employment. Initially, all 19 categories were used to assess the prevalence of problem drinking (PPD) across industries.

Afterwards, these categories were grouped into seven gender-typed industry categories based on two principles: (1) the main job activities of the industry, and (2) the proportion of men and women employed in the industry, following the methodology outlined by Härenstam and Nyberg [13] and Cerdas et al. [23]. First, the 19 SNI categories were grouped into seven broader industry categories based on the main job activities. Härenstam and Nyberg argued that the type of industry production or main job activity would reflect differences in organizational factors [13]. Based on this argument, they separated industries where work is mainly place-bound activities in factories or mines, from those where work is mainly performed in different places and still utilizes the machinery and vehicles that have male connotations. These two groups were levelled “Goods and Energy Production” and “Machinery Operations”. Similarly, two major SNI 2007 categories were classified separately as “Education” and “Health and Social Care”. Additionally, service-oriented and administrative activities based SNI industries were categorised as either “Labour Intensive Services” or “Knowledge Intensive Services” as they are associated with class rather than gender. However, one of the main SNI categories, Public Administration, was not an easy fit for any of these categories and was kept in a separate “Public Administration” category that includes both knowledge-intensive public administration and activities like military, police and fire brigades. Secondly, these seven industry categories were classified as either female-dominated (Health and Social Care, Education), gender-mixed (Public Administration, Labour Intensive Services, and Knowledge Intensive Services) or male-dominated (Goods and Energy Production, and Machinery Operations) industries, depending on the proportion of men and women employed in these categories. If at least 70% of the people employed in the industry were male or female, the industry was typed as male or female dominated. Otherwise, it was classified as gender-mixed industry. The main job activity of female-dominated industries was interacting with people, while in male-dominated industries, it was handling things and in gender-mixed industries, the main activities were handling things, data and people.

For the present study, the seven industry categories were included in the regression models as a proxy of gender-typed industries to examine whether problem drinking patterns differed based on gender composition and main job activities. Table 1 provides a detailed summary of the industry categorisation of our study population and the percentage of female workers in both the original SNI 2007 and gender-typed industry categories.

**Table 1:** Industry Classification of Study Population (*n* = 9155) by Gender Composition and Main Job Activity, 2020

Industry Categories	SNI 2007 categories and code	% of female workers	Main job activity	Gender-based Categories
**Health and Social Care**		**85.4%**	People	Female-dominated
	Human health and social work activities (Q)	85.4%		
**Education**		**79.7%**	People	Female-dominated
	Education (P)	79.7%		
**Public Administration**		**67.2%**	People Data	Gender-mixed
	Public administration and defence; compulsory social security (O)	67.2%		
**Labour Intensive Services**		**55.7%**	People Things Data	Gender-mixed
	Wholesale and retail trade, repair of motor vehicles and motorcycles (G)	47.5%		
	Accommodation and food service activities (I)	56.3%		
	Arts, entertainment, and recreation (R)	64.2%		
	Other service activities (S)	69.4%		
	Administrative and support service activities (N)	59.6%		
**Knowledge Intensive** **Services**		**43.7%**	Data	Gender-mixed
	Information and communication (J)	33.8%		
	Financial and insurance activities (K)	56.3%		
	Real estate activities (L)	40.7%		
	Professional, scientific, and technical activities (M)	46.1%		
**Goods and Energy Production**		**30.0%**	Things	Male-dominated
	Mining and quarrying (B)	15.4%		
	Manufacturing (C)	29.7%		
	Electricity, gas, and air conditioning supply (D)	37.4%		
	Water supply; sewerage, waste management, and remediation activities (E)	25.0%		
**Machinery Operations**		**21.3%**	Things	Male-dominated
	Agriculture, forestry, and fishing (A)	34.6%		
	Construction (F)	13.4%		
	Transportation and storage (H)	28.0%		

#### Problem drinking

Many terms have been used to describe the misuse of alcohol. The term used in this study (problem drinking) refers to alcohol use that has caused problems but can be both on a clinical or subclinical level and detectable by the Cut-down, Annoyed, Guilt, Eye-opener (CAGE) tool [26–28]. To identify potential problem drinking cases, participants were asked to answer a slightly modified version of the CAGE questionnaire, a four-question alcohol screening tool [29]. The original CAGE questionnaire, which begins each question with “Have you ever” to indicate lifetime risk, was modified by removing the word “ever” to focus on more recent drinking problems that the participants might have experienced, rather than including all possible experiences of problem drinking during the participants’ lifetime. CAGE is well-validated in different populations and it is a highly effective tool for detecting problem drinking with 93% sensitivity and 76% specificity [30]. In this questionnaire, participants answer four yes-no questions, scoring one point for each “yes” and zero point for each “no”. Generally, a total score of 2 or higher indicates a possible case of problem drinking [30, 31]. In this study, we used a score of 2 to identify possible cases and created a binary outcome variable labelled “problem drinking,” coding individuals with a CAGE score of ≥ 2 as “Problem Drinking” (1) and those with a score < 2 as “No Problem Drinking” (0).

#### Covariates

Participants’ ages were divided into five categories (≤ 35, 36–45, 46–55, 56–65, ≥ 65) to reflect the various phases of individuals’ working lives. Given that the retirement age in Sweden ranges from 63 to 69 years, and that an increasing number of people are choosing to work beyond this age, we decided not to restrict study participation to individuals aged 65 and below [32]. This choice allowed us to include this new working demographic in our study. Self-employed individuals were excluded from the study to focus solely on employees. Participants’ highest levels of education were classified into five categories: compulsory education (≤ 9 years), upper secondary education (2 years), upper secondary educaton (3-4 years), university education (< 3 years), and university education (≥ 3 years). Civil status was categorised into two groups: living without a partner and living with a partner.

### Statistical analysis

Descriptive statistics were used to characterise the study population and assess the prevalence of problem drinking across industries. Differences in problem drinking prevalence among different covariates were analysed using Chi-square test or Fisher’s exact test when appropriate. Poisson regression with robust standard errors (SE), using the heteroscedasticity-consistent covariance matrix estimator method (HC1), was used to investigate the association between gender-typed industries (exposure) and problem drinking (outcome). This method, an alternative to logistic regression, allows for direct estimation of prevalence ratios (PR) via the exponential function of the Poisson model coefficient and enables the calculation of associated 95% confidence intervals (CI) for each industry category [33, 34]. The industry with the lowest prevalence of problem drinking was selected as the reference category in the regression analysis. We constructed two models: Model 1 (crude) included gender-typed industries as the sole predictor, while Model 2 was adjusted for potential confounders such as age, civil status, education, and gender. To further explore gender-specific associations, we conducted a gender-stratified analysis adjusting for age, civil status and level of education within each gender stratum.

Additionally, we conducted a sensitivity analysis by reclassifying industries into three broader gender-based industry categories: female-dominated, gender-mixed, and male-dominated based only on gender composition of industries, without considering the main job activity. SNI 2007 industries with more than 70% of the workforce belonging to one gender were categorised as either female-dominated or male-dominated while the remaining industries were categorised as gender-mixed industries. This reclassification allowed us to assess the robustness of our findings and determine whether associations varied when considering only the gender composition of the industries. To further investigate the strength of the findings, we conducted additional sensitivity analyses by redefining the outcome variable (problem drinking), using CAGE cut-off 1. All statistical analyses were conducted using R software (version 4.3.2 or higher).

## Results

### Cohort characteristics

Tables 2 and 3 provide a comprehensive overview of study participants, including a breakdown of their work industry and prevalence of problem drinking in each category. Among the participants, 58% were female, and over 75% were 46–65 years old. 59% were married or living with a partner, and 51% had a university-level education. The largest work industry for the participants was Human health and social work activities (19.4%), followed by Education (14.7%), Manufacturing (12.0%), Public administration and defence; compulsory social security (11.3%), and Wholesale and retail trade; repair of motor vehicles and motorcycles (8.1%). Gender-wise, the primary work industry for men was Manufacturing (20.0%), while for women it was Human health and social work activities (28.6%).

**Table 2:** Prevalence of Problem Drinking in Study Population Stratified by Gender, 2020

Problem Drinking	Total (*n* = 9155)			Men (*n* = 3857)			Women (*n* = 5298)			Difference
	*n*	%	95% CI	*n*	%	95% CI	*n*	%	95% CI	*P*-value
										< 0.001
No	8547	93.4%	92.8–93.8	3531	91.5%	90.6–92.4	5016	94.7%	94.0–95.2	
Yes	608	6.6%	6.2–7.2	326	8.5%	7.6–9.4	282	5.3%	4.8–6.0	

*P-value from the chi-square test*

**Table 3:** Covariate Distribution and Prevalence of Problem Drinking (PPD) by Gender in Study Population, 2020

	Total				Men				Women			
	*n*	%	PPD (95% CI)	*P*-value	*n*	%	PPD (95% CI)	*P*-value	*n*	%	PPD (95% CI)	*P*-value
**Age**				0.20				0.68				0.29
≤ 35	327	3.6%	4.6% (2.8–7.4)		116	3.0%	7.8% (4.1–14.1)		211	4.0%	2.8% (1.3–6.1)	
36–45	1507	16.4%	5.7% (4.6-7.0)		594	15.4%	7.6% (5.7–10.0)		913	17.2%	4.5% (3.3-6.0)	
46–55	3221	35.2%	6.8% (6.0-7.7)		1365	35.4%	8.0% (6.7–9.5)		1856	35.0%	5.9% (4.9-7.0)	
56–65	3663	40.0%	7.0% (6.2–7.8)		1536	39.8%	9.1% (7.7–10.6)		2127	40.2%	5.5% (4.6–6.5)	
≥ 65	437	4.8%	7.8% (5.6–10.7)		246	6.4%	9.8% (6.6–14.1)		191	3.6%	5.2% (2.9–9.4)	
**Civil Status**				0.18				0.47				0.19
Living without partner	3747	40.9%	7.1% (6.3–7.9)		1531	39.7%	8.9% (7.6–10.4)		2216	41.8%	5.8% (4.9–6.9)	
Living with partner	5408	59.1%	6.3% (5.7-7.0)		2326	60.3%	8.2% (7.1–9.4)		3082	58.2%	5.0% (4.3–5.8)	
**Education**				0.48				0.03				0.11
Compulsory school (≤ 9 years)	536	5.9%	5.8% (4.1–8.1)		266	6.9%	6.8% (4.3–10.4)		270	5.1%	4.8% (2.8–8.1)	
Upper secondary (2 years)	1734	18.9%	7.2% (6.0-8.5)		983	25.5%	9.9% (8.2–11.9)		751	14.2%	3.6% (2.5–5.2)	
Upper secondary (3-4 years)	2217	24.2%	7.1% (6.1–8.3)		1020	26.4%	8.7% (7.1–10.6)		1197	22.6%	5.8% (4.6–7.2)	
University (< 3 years)	1353	14.8%	6.7% (5.5–8.2)		501	13.0%	10.2% (7.8–13.1)		852	16.1%	4.7% (3.5–6.3)	
University (≥ 3 years)	3315	36.2%	6.2% (5.4-7.0)		1087	28.2%	6.5% (5.2–8.2)		2228	42.0%	6.0% (5.1-7.0)	
**Work Industry**				0.00				0.73				0.24
Accommodation and food service activities	80	0.9%	10.0% (5.2–18.5)		35	0.9%	11.4% (4.5–26.0)		45	0.8%	8.9% (3.5–20.7)	
Administrative and support service activities	240	2.6%	8.3% (5.5–12.5)		97	2.5%	9.3% (5.0-16.7)		143	2.7%	7.7% (4.4–13.2)	
Agriculture, forestry, and fishing	81	0.9%	4.9% (1.9–12.0)		53	1.4%	5.7% (1.9–15.4)		28	0.5%	3.6% (0.6–17.7)	
Arts, entertainment, and recreation	165	1.8%	5.5% (2.9–10.0)		59	1.5%	5.1% (1.7–13.9)		106	2.0%	5.7% (2.6–11.8)	
Construction	402	4.4%	8.5% (6.1–11.6)		348	9.0%	8.1% (5.6–11.4)		54	1.0%	11.1% (5.2–22.2)	
Education	1348	14.7%	6.8% (5.5–8.2)		274	7.1%	9.1% (6.3–13.1)		1074	20.3%	6.2% (4.9–7.7)	
Electricity, gas, steam, and air conditioning supply	99	1.1%	5.1% (2.2–11.3)		62	1.6%	6.5% (2.5–15.4)		37	0.7%	2.7% (0.4–13.8)	
Financial and insurance activities	229	2.5%	10.0% (6.8–14.6)		100	2.6%	13.0% (7.8–21.0)		129	2.4%	7.8% (4.3–13.7)	
Human health and social work activities	1772	19.4%	4.7% (3.9–5.8)		259	6.7%	8.1% (5.4–12.1)		1513	28.6%	4.2% (3.3–5.3)	
Information and communication	397	4.3%	6.3% (4.3–9.1)		263	6.8%	7.2% (4.7–11.0)		134	2.5%	4.5% (2.1–9.4)	
Manufacturing	1098	12.0%	8.0% (6.6–9.8)		772	20.0%	8.6% (6.8–10.7)		326	6.2%	6.8% (4.5–10.0)	
Mining and quarrying	13	0.1%	15.4% (4.3–42.2)		11	0.3%	18.2% (5.1–47.7)		2	0.0%	0	
Other service activities	271	3.0%	5.9% (3.7–9.4)		83	2.2%	10.8% (5.8–19.3)		188	3.5%	3.7% (1.8–7.5)	
Professional, scientific, and technical activities	664	7.3%	6.2% (4.6–8.3)		358	9.3%	7.5% (5.2–10.8)		306	5.8%	4.6% (2.7–7.5)	
Public administration and defence; compulsory social security	1037	11.3%	6.1% (4.6–8.3)		340	8.8%	7.7% (5.3–11.0)		697	13.2%	5.3% (3.9–7.2)	
Real estate activities	162	1.8%	11.1% (7.1–16.9)		96	2.5%	12.5% (7.3–20.6)		66	1.2%	9.1% (4.2–18.4)	
Transportation and storage	314	3.4%	5.4% (3.4–8.5)		226	5.9%	6.6% (4.1–10.7)		88	1.7%	2.3% (0.6–7.9)	
Water supply; sewerage, waste management and remediation activities	44	0.5%	2.3% (0.4–11.8)		33	0.9%	3.0% (0.5–15.3)		11	0.2%	0	
Wholesale and retail trade; repair of motor vehicles and motorcycles	739	8.1%	8.0% (6.2–10.2)		388	10.1%	10.1% (7.4–13.4)		351	6.6%	5.7% (3.7–8.6)	

*P-value from Chi-square or Fisher’s exact test*,* as appropriate*

*PPD = Prevalence of Problem Drinking*

### Prevalence of problem drinking (PPD)

As presented in Table 2, the prevalence of problem drinking was 6.6% in the study population. Men had a higher prevalence (8.5%) than women (5.3%) and this difference in prevalence was statistically significant.

Among the age groups, individuals aged 65 and older had the highest prevalence of problem drinking (7.8%). Gender-wise, men aged 65 years or older had the highest prevalence (9.8%), while for women the highest prevalence was in the 46–55 age group (5.9%). Individuals living alone had a higher prevalence (7.1%) compared to those living with a partner (6.3%), a pattern observed in both genders. Additionally, participants with the lowest level of education (compulsory school) had the lowest prevalence (5.8%) of problem drinking. However, in terms of gender, men with three years or higher university level education had the lowest prevalence (6.5%), whereas for women the lowest prevalence (3.6%) was in the two years secondary education category. Despite these, the differences in prevalence across age groups, civil status, and education levels were not statistically significant in the study population according to the Chi-square test. However, gender-wise, for women prevalence differences were not significant in age groups, civil status and education level, whereas for men, only differences in prevalence across education categories were significant.

Across industries, the highest prevalence of problem drinking was observed in Mining and quarrying (15.4%), followed by Real estate activities (11.1%), Accommodation and food service activities (10.0%), Financial and insurance activities (10.0%), and Construction (8.5%). On the other hand, the lowest prevalence was observed in Water supply, sewerage, waste management, and remediation activities (2.3%), followed by Human health and social work activities (4.7%), Agriculture, forestry, and fishing (4.9%), and Electricity, gas, steam, and air conditioning supply (5.1%).

For men, the highest prevalence of problem drinking was in Mining and quarrying (18.2%), and the lowest was in Water supply, sewerage, waste management, and remediation activities (3.0%). In contrast, for women, the highest prevalence was observed in Construction (11.1%), and the lowest in Mining and quarrying (0), and Water supply; sewerage, waste management and remediation activities (0). Nevertheless, the differences in prevalence across industries for both men and women were not statistically significant according to Fisher’s exact test.

### Problem drinking in industries based on gender composition and main job activity

Table 4 presents the prevalence of problem drinking in gender-typed industries. The highest prevalence of problem drinking was observed in Goods and Energy Production (7.7%), followed by Labour-intensive Services (7.5%), and Knowledge-intensive Services (7.4%). Goods and energy production is a male-dominated industry while both Labour-intensive Services and Knowledge-intensive Services are gender-mixed industries (see Table 1). In contrast, the lowest prevalence was observed in Health and Social Care (4.7%), which is a female-dominated industry.

**Table 4:** Prevalence of Problem Drinking (PPD) in Gender-typed Industries in Study Population, 2020

Gender-Typed Industry Categories	Total				Men				Women			
	*n*	%	PPD (95% CI)	*P*-value	*n*	%	PPD (95% CI)	*P*-value	*n*	%	PPD (95% CI)	*P*-Value
				0.01				0.83				0.38
**Female Dominated**												
Health & Social Care	1772	19.4%	4.7% (3.9–5.8)		259	6.7%	8.1% (5.4–12.1)		1513	28.6%	4.2% (3.3–5.3)	
Education	1348	14.7%	6.8% (5.5–8.2)		274	7.1%	9.1% (6.3–13.1)		1074	20.3%	6.2% (4.9–7.7)	
**Gender mixed**												
Public Administration	1037	11.3%	6.1% (4.8–7.7)		340	8.8%	7.7% (5.3–11.0)		697	13.2%	5.3% (3.9–7.2)	
Labour Intensive Services	1495	16.3%	7.5% (6.3–8.9)		662	17.2%	9.7% (7.6–12.2)		833	15.7%	5.8% (4.4–7.6)	
Knowledge Intensive Services	1452	15.9%	7.4% (6.1–8.8)		817	21.2%	8.7% (7.0-10.8)		635	12.0%	5.7% (4.1–7.8)	
**Male Dominated**												
Goods and Energy Production	1254	13.7%	7.7% (6.3–9.3)		878	22.8%	8.3% (6.7–10.3)		376	7.1%	6.1% (4.1-9.0)	
Machine Operation	797	8.7%	6.9% (5.3–8.9)		627	16.3%	7.3% (5.6–9.7)		170	3.2%	5.3% (2.8–9.8)	

*P-value from Chi-square test*

*PPD = Prevalence of Problem Drinking*

For men, the highest prevalence of problem drinking was in Labour-intensive Services (9.7%), which is a gender-mixed industry, while the lowest prevalence was observed in Machine Operations (7.3%), a male-dominated industry. For women, the highest prevalence was in Education industry (6.2%), while the lowest prevalence was in Health and Social Care industry (4.2%). Despite these observations, the Chi-square test did not find any significant differences in prevalence across gender-typed industry categories either for men or women.

Table 5 describes the result of the Poisson regression model in both unadjusted and adjusted models. In the unadjusted model (M1), there are indications for a higher risk of prevalence of problem drinking in the Education industry (PR: 1.42, *p* = 0.016), Labour-intensive Services (PR: 1.58, *p* = 0.001), Knowledge-intensive Services (PR: 1.56, *p* = 0.002), Goods and Energy Production (PR: 1.62, *p* = 0.001) and in Machinery Operations (PR: 1.46, *p* = 0.026) in comparison with Health and Social Care industry. However, in the adjusted model (M2), only the Education industry (aPR: 1.39, *p* = 0.026) and Labour-intensive Services (aPR: 1.39, *p* = 0.022) showed statistically significant higher adjusted prevalence ratios (aPR).

**Table 5:** Poisson Regression Analysis with Robust Standard Errors for Problem Drinking in Gender-typed Industries

Gender-typed Industry Categories	M1		M2	
	PR (95% CI)	*P*-value	aPR (95% CI)	*P*-value
**Female Dominated**				
Health and Social Care	Ref		Ref	
Education	1.42 (1.07–1.90)	0.016	1.39 (1.04–1.86)	0.026
**Gender mixed**				
Public Administration	1.28 (0.93–1.76)	0.126	1.19 (0.86–1.65)	0.282
Labour Intensive Services	1.58 (1.20–2.08)	0.001	1.39 (1.05–1.85)	0.022
Knowledge Intensive Service	1.56 (1.18–2.05)	0.002	1.31 (0.98–1.76)	0.069
**Male Dominated**				
Goods and Energy Production	1.62 (1.22–2.15)	0.001	1.28 (0.94–1.57)	0.118
Machinery Operations	1.46 (1.05–2.02)	0.026	1.11 (0.78–1.59)	0.561

M1 – Unadjusted model, M2 - Adjusted for age, civil status, education, and gender

PR = Prevalence Ratio, aPR = Adjusted Prevalence Ratio

Table 6 presents adjusted prevalence ratios (aPRs) with 95% confidence intervals (CIs) for problem drinking, with separate analyses conducted for each gender within gender-typed industry categories. The stratified analysis revealed that women in both Education (aPR: 1.43, *p* = 0.04) and Labour-intensive Services (aPR: 1.45, *p* = 0.05) had significantly higher prevalence compared to the reference category Health and Social Care. Additionally, there was a trend suggesting possible increased prevalence in Goods and Energy Production (aPR: 1.55, *p* = 0.07) compared to the reference category. Conversely, no significant differences in problem drinking were observed among men across gender-typed industries.

**Table 6:** Gender-stratified Poisson Regression Analysis with Robust Standard Errors for Problem Drinking in Gender-typed Industries

Gender-typed Industry Categories	Men		Women	
	aPR (95% CI)	*P*-value	aPR (95% CI)	*P*-value
**Female Dominated**				
Health and Social Care	Ref		Ref	
Education	1.16 (0.66–2.02)	0. 61	1.43 (1.02–2.01)	0.04
**Gender mixed**				
Public Administration	0.90 (0.51–1.59)	0.72	1.28 (0.86–1.90)	0.23
Labour Intensive Services	1.08 (0.67–1.74)	0.74	1.45 (1.00-2.10)	0.05
Knowledge-intensive Service	1.04 (0.65–1.67)	0.86	1.38 (0.92–2.07)	0.11
**Male Dominated**				
Goods and Energy Production	0.92 (0.57–1.48)	0.74	1.55 (0.97–2.48)	0.07
Machinery Operations	0.78 (0.47–1.29)	0.33	1.35 (0.68–2.67)	0.39

Adjusted for age, civil status, education

aPR = Adjusted Prevalence Ratio

### Sensitivity analysis

To test the strength and robustness of our findings we conducted three different sensitivity analyses. Firstly, we redefined the outcome variable by using CAGE score ≥ 1 and used it in the regression model for both a total and a gender-stratified analysis. In the overall model, Education remained significant (aPR: 1.20, *p* = 0.03), whereas Labour Intensive Services, which was significant in the primary analysis, lost its significance (aPR: 1.04, *p* = 0.63) and Knowledge Intensive Services which showed a trend in the primary analysis became significant (aPR: 1.21, *p* = 0.02). From the gender stratified analysis, there were no significant changes for men. However, for women, there were some notable changes, for example, Education which was significant in the main analysis became a trend (aPR: 1.18, *p* = 0.09) and both Labour Intensive Services and Goods and Energy Production, which showed a trend in the main analysis, now weakened and no longer a trend.

Additionally, we repeated the regression analysis using reclassified gender-typed industries with three categories (male-dominated, gender-mixed and female-dominated) as a predictor variable. This approach also did not find any statistically significant difference in gender-mixed industries or male-dominated industries compared to female-dominated industries. However, when considering CAGE score ≥ 1 to define problem drinking and investigating it against three gender-typed industry categories, men working in male-dominated industries showed a protective trend (aPR: 0.84, *p*: 0.07) compared with female-dominated industries. No other changes were noted in the findings. The results of the sensitivity analyses are available in Appendix I, II and III.

## Discussion

In this study, we assessed the prevalence of problem drinking in employees in various industries in the Swedish workforce. We analysed prevalences according to (1) the Swedish Standard Industrial Classification (19 industries), (2) industries categorized by gender composition and main job activity (7 categories), and (3) gender composition only (3 categories).

It is important to note that some industries like Accommodation and food service activities, Agriculture, forestry, and fishing, Mining and quarrying, and Water supply; sewerage, waste management and remediation activities had relatively small sample sizes, which could influence the reliability of the prevalence estimates and require cautious interpretation. Despite this, the Mining and quarrying industry had the highest prevalence of problem drinking, followed by Real estate, Accommodation and food service activities, Financial and insurance activities, and Construction.

In comparisons between seven gender-typed industry categories based on gender composition and main job activity, problem drinking was higher in the Education industry and in Labour-intensive Services compared with the Health and Social Care industry. There were no differences in problem drinking between industries when categorised into female-dominated, male-dominated, and gender-mixed.

### Differences between the 19 industries based on the Swedish Standard Industrial Classification

While the small sample sizes in some industry categories based on the SNI 2007 classification and wide confidence intervals (CIs) of the prevalence estimates limit the precision of our estimates, particularly for Mining and quarrying (PPD: 15.4%, CI: 4.3–42.2) and Accommodation and food service activities (PPD: 10.0%, CI: 5.2–18.5), these industries have previously been recognized as high-risk industries for problem drinking. For instance, studies from Australia [35, 36], Canada [37], and the USA [38] have consistently reported high alcohol consumption among mine workers. Similarly, other studies also mentioned high alcohol consumption in Accommodation and food service industries (also known as the Hotel and restaurant industry) due to various factors including easy access to alcohol and workplace cultural dynamics [39–41]. Our findings are therefore consistent with existing knowledge.

Prior studies in Sweden on this topic have mainly focused on occupational gender composition [20] or focused on single industries [22], limiting the possibility of direct comparisons. However, a US-based study investigating heavy alcohol use among current workers across industries from 2003 to 2012 found that Mining, Construction, Arts, Entertainment and recreation, Accommodation and food services, and Wholesale had the highest alcohol consumption in the workforce [38]. Additionally, an Australian study based on National Drug Strategy Household Survey 2001 reported more frequent alcohol consumption in Agriculture, Retail, Wholesale, Hospitality, Manufacturing, Construction, and Transport industries [41]. These findings are similar to ours and suggest that differences in drinking patterns between labour market industries may be similar in western industrialized countries.

An interesting finding from our study is that the Real estate industry had the second-highest prevalence of problem drinking (PPD: 11.1%, CI: 7.1–16.9), surpassing the traditionally high-risk Construction industry (PPD: 8.5%, CI: 6.1–11.6). Several explanations might account for this result. Historically, the construction sector has been identified as a high-risk industry for alcohol-related problems, leading to numerous interventions and awareness activities targeting this industry. This could explain the relatively low prevalence observed in this industry. Additionally, our study focused on current employees and used CAGE cut-off 2 to detect problem drinking, which might have missed some early-stage cases of problem drinking, due to its lower sensitivity to early-stage cases [42]. It is possible that stricter controls in high-risk industries like Construction might be better at identifying initial signs of problematic alcohol use and removing affected employees from the workplace, leading to a lower observed prevalence in this industry. Conversely, higher prevalence in service sectors like real estate or finance and insurance may be linked to different psychosocial work factors, such as a culture of celebration, work-related socialisation, or job-related stressors like income insecurity or poor work-life balance etc. The high prevalence of problem drinking in some underexplored industries like Real estate and Financial and insurance activities warrants further close investigation.

Nevertheless, since a CAGE score of ≥ 2 was used to measure the prevalence of problem drinking in general, it is important to mention that, it is less sensitive in detecting early-stage drinking problems and may underestimate problem drinking among women [42, 43]. As a result, our measured prevalence estimates are likely conservative and should be interpreted with caution. However, to mitigate any potential impact of this conservative measurement of problem drinking on our findings, we conducted an additional sensitivity analysis by using CAGE cut-off 1. These findings are discussed in the following section.

### Differences among the seven industries based on gender composition and main job activity

To the best of our knowledge, this study is among the first to investigate the association between gender-typed industries and employee problem drinking. The results from the unadjusted model (M1) showed statistically significant differences in prevalence across most gender-typed industries except Public Administration. However, in the adjusted model (M2), only Education and Labour-intensive Services remained statistically significant, with a 39% higher prevalence of problem drinking in both industry categories compared to Health and Social Care. Interestingly, both Education and Health and Social Care are female-dominated industries, and their main job activities involve dealing with people. This difference between two female-dominated industries suggests that industry-specific psychosocial factors may be more important than the gender composition of industries in determining employee problem drinking. Additionally, the results from the sensitivity analysis (Appendix I) by using CAGE cut-off 1 which is sensitive to less severe or early-stage cases of problem drinking also do not change the primary direction of the findings that much. In the sensitivity analysis, the difference between Education and Health and Social Care remains significant, though Labour Intensive Services loses its significance and Knowledge Intensive Services becomes significant. However, this shift in results in some industry categories suggests possible differences in alcohol consumption patterns across industries and further research is needed to explore underlying factors contributing to this variation.

The gender-stratified analysis found that women working in Education have a 43% higher prevalence of problem drinking than those in Health and Social Care. This significance however weakens and becomes a trend, and the previously displayed trend in Goods and Energy Production disappears when considering more liberal measures of problem drinking in the sensitivity analysis (Appendix I). On the other hand, for men, there were no significant associations for problem drinking across gender-typed industries (Table 6) and stayed the same in the sensitivity analysis. These different results from the gender-stratified analysis for men and women indicate that industry-specific factors might play a more important role in shaping problem drinking among women than men.

### Differences between industries based on gender composition only

The sensitivity analysis, which considered only the gender composition of the industries while ignoring the job activities, did not find any significant difference in the risk of problem drinking between male-dominated, gender-mixed, and female-dominated industries. Additionally, the gender-stratified analysis did not reveal any associations for either men or women, reaffirming the absence of any significant association between gender-typed industries and problem drinking. Moreover, an additional sensitivity analysis using CAGE cut-off 1 to define problem drinking also showed no association, further strengthening our findings. However, in the gender-stratified analysis, there is a trend suggesting a possible protective effect against problem drinking for men working in male-dominated industries, which requires further investigation.

Most existing studies on this topic (from Sweden [20, 22], Finland [19], Canada [44], the US [18, 45], the UK [46] and Spain [47]) have focused on occupational gender composition and problem drinking. Therefore, it is not possible to directly compare most previous results with our findings. However, one study from the US explored the drinking patterns of female workers based on both gender composition of industries and occupations and reported an increased risk of alcohol consumption among women in gender-mixed industries [21]. In our gender-stratified analysis, we also observed an increasing trend of problem drinking for women workers in gender-mixed and male-dominated industries compared to female-dominated industries. However, the result was not statistically significant.

Though the study from the US [21] reported a curvilinear relationship between the percentage of men in any given industry and women’s drinking, the findings of this study are based on data from the 1990s and may not reflect current drinking trends, as drinking norms, culture, and work-life factors change over time [48, 49]. Therefore, further research with contemporary data would be required to capture current drinking patterns in the US workforce. Our findings, which utilize recent industry-specific data and systematically categorise all industries based on gender composition and main job activity, are likely to provide a more accurate representation of current drinking patterns in the Swedish workforce.

### Strengths and limitations

The main strength of this study is the use of an industry-specific categorization based on both gender composition of the industries and job activities rather than on occupation-based categories, which provides a more precise reflection of work-life dimensions on problem drinking. Additionally, this is one of the first studies to measure industry-specific prevalences of problem drinking in the Swedish workforce and could guide industry-specific future research. Moreover, this study utilised both self-reported and population registers data, which reduces the potential risk of self-reported bias on some variables.

One potential limitation of this study is the overrepresentation of highly educated, professional and older workers in our study population. According to Statistics Sweden’s (SBC) Labour Market Report 2021, the proportion of men was 53% in the Swedish labour market, while women constituted 47% [50]. In contrast, our study sample was comprised of 58% women. Additionally, another report by SBC indicates that approximately 44% of the general population aged between 25 and 64 years had some kind of university-level education in 2020 [51]. However, this group amounted to 51% of the sample population of this study. While these characteristics of study participants could introduce bias into our findings, the use of industry categories instead of professional groups ensured that individuals with varying educational backgrounds, professional categories and ages were distributed across different industries, potentially mitigating this concern. Furthermore, the response rate for the SLOSH 2020 wave was 49% for both working and out-of-work participants and had a relatively high representation of female workers, public sector employees, and Swedish-born participants in the study sample, which limits the generalisability of our findings and requires caution in interpreting the results.

Another potential limitation is that alcohol consumption patterns were self-reported using the CAGE tool, which means there is both a risk of the healthy worker effect and that the prevalence of problem drinking could be underestimated. While a CAGE score of ≥ 2 is conventionally used to identify alcohol-related problems, some researchers have suggested to use a score of ≥ 1 for general screening purposes, arguing that this would increase the tool’s sensitivity and improve the detection of early-stage cases [43]. However, a meta-analysis of CAGE studies concluded that a cut-off of 2 is more appropriate, as using a cut-off of 1 significantly reduces the tool’s specificity, thereby increasing the risk of false positives [52]. To address this concern and test the robustness of our findings, we conducted sensitivity analyses using a CAGE cut-off 1 to define possible problem drinking cases. Though this analysis did not alter the primary findings, we observed some shifts in significance in the seven industry categories and some previously significant results for women became insignificant (see Appendix I & III). These small variations indicate that our findings may be influenced by our choice of CAGE cut-off for defining problem drinking, and the use of a lower threshold to define problem drinking could potentially reveal different patterns of risk, especially for certain subgroups.

Additionally, the SLOSH data used in this study were collected during the early stages of the COVID-19 pandemic, which could potentially have influenced data collection or study findings. However, it is important to note that Sweden was one of few countries that did not implement strict social measures or restrictions during the pandemic [53]. Moreover, a recent study found no significant change in alcohol use in Sweden during the pandemic period [54]. This suggests that the impact of the COVID-19 pandemic on our study is likely negligible.

Another potential limitation is the inherent inability of a cross-sectional design to establish causal relationships or track changes in drinking patterns over time. However, this limitation is not a major concern given that the focus of this study is not on determining causal relationships, but solely on identifying the prevalence of problem drinking and differences in prevalence in different industry categories.

## Conclusion

Our study revealed that the prevalence of problem drinking in the Swedish workforce is not uniform across industries, with notable differences observed between men and women. Overall, men had a higher prevalence of problem drinking than women. Additionally, there were differences in the risk of problem drinking across industries categorised by gender composition and main job activity, but not when categorised into gender composition only. Industry-specific psychosocial work environment dynamics may have contributed to the observed problem drinking patterns. Future research should investigate industry-specific psychosocial work environment factors (e.g., job stress, work-life balance) and their potential impacts on problem drinking patterns within the workforce.

## Electronic supplementary material

Below is the link to the electronic supplementary material.

Additional file 1: Tables (Appendix I, Appendix II & Appendix III)

## Data Availability

SLOSH dataset supporting the conclusions of this article is available to researchers under special request. Detailed information about the SLOSH cohort and how to obtain the SLOSH data can be found at https://slosh.se/for-researchers-in-english/introduction.

## Abbreviations

PPD: Prevalence of Problem Drinking
PR: Prevalence Ratio
aPR: Adjusted Prevalence Ratio
SLOSH: Swedish Longitudinal Occupational Survey of Health
CAGE: Cut-down, Annoyed, Guilt, Eye-opener questionnaire
95% CI: 95% Confidence Intervals
