# Supplementary material for: Prevalence of problem drinking in the Swedish workforce: differences between labour market industries based on gender composition and main job activity
Source: BMC Public Health. 2024 Oct 1;24:2683. doi: 10.1186/s12889-024-20163-y (PMC11443882; doi:10.1186/s12889-024-20163-y)
Supplement: Supplementary file 1 — Additional file 1: Tables (Appendix I, Appendix II & Appendix III) [file 12889_2024_20163_MOESM1_ESM.docx]

**Appendix I**

**Sensitivity analysis 1: Sensitivity analysis by using CAGE cutoff 1 to define problem drinking in the study population**

Table 7: Gender-Stratified Poisson Regression Analysis with Robust Standard Errors for Problem Drinking Using CAGE Cutoff 1 in Gender-typed Industries

| Gender-typed Industry Categories | Total^*^ | | Men^***^ | | Women^***^ | |
| --- | --- | --- | --- | --- | --- | --- |
|  | aPR (95% CI) | P-value | aPR (95% CI) | P-value | aPR (95% CI) | P-value |
| **Female Dominated** |  |  |  |  |  |  |
| Health and Social Care | Ref |  | Ref |  | Ref |  |
| Education | 1.20 (1.02-1.41) | 0.03 | 1.17 (0.86-1.59) | 0.32 | 1.18 (0.98-1.43) | 0. 09 |
| **Gender mixed** |  |  |  |  |  |  |
| Public Administration | 1.15 (0.97-1.37) | 0.11 | 1.11 (0.82-1.50) | 0.50 | 1.14 (0.92-1.43) | 0.23 |
| Labour Intensive Services | 1.04 (0.88-1.23) | 0.63 | 0.99 (0.75-1.30) | 0.92 | 1.03 (0.82-1.29) | 0.78 |
| Knowledge-intensive Service | 1.21 (1.04-1.42) | 0.02 | 1.17 (0.90-1.51) | 0.25 | 1.21 (0.96-1.51) | 0.10 |
| **Male Dominated** |  |  |  |  |  |  |
| Goods and Energy Production | 0.99 (0.83-1.19) | 0.94 | 0.88 (0.67-1.16) | 0.37 | 1.13 (0.85-1.51) | 0.39 |
| Machinery Operations | 1.01 (0.83-1.24) | 0.88 | 0.88 (0.66-1.18) | 0. 39 | 1.21 (0.82-1.79) | 0.33 |
| ^*^Adjusted for age, civil status, education, and Gender; ^***^Adjusted for age, civil status, and education  aPR = Adjusted Prevalence Ratio | | | | | | |

**Appendix II:**

**Sensitivity analysis 2: Sensitivity analysis by categorising work industries of the study population into three gender-typed categories based on gender composition only.**

Table 8: Gender Stratified Poisson Regression Analysis with Robust Standard Errors for Problem Drinking in three Gender-typed Industries

| Gender typed Industries | Total | | Men | | Women | |
| --- | --- | --- | --- | --- | --- | --- |
|  | aPR (95% CI) | P- Value | aPR (95% CI) | P-Value | aPR (95% CI) | P-Value |
| Female-Dominated | Ref |  | Ref |  | Ref |  |
| Gender-mixed | 1.11 (0.92-1.35) | 0.29 | 0.95 (0.69-1.30) | 0.73 | 1.15 (0.91-1.47) | 0.25 |
| Male-dominated | 1.05 (0.82-1.35) | 0.70 | 0.80 (0.56-1.14) | 0.22 | 1.32 (0.90-1.95) | 0.16 |

*Adjusted for age, civil status, and education*

*aPR = Adjusted Prevalence Ratio*

**Appendix III:**

**Sensitivity analysis 3: Sensitivity analysis using CAGE cutoff 1 to define problem drinking and categorising work industries of the study population into three gender-typed categories based on gender composition only.**

Table 9: Gender Stratified Poisson Regression Analysis with Robust Standard Errors for Problem Drinking Using CAGE Cutoff 1 in three Gender-typed Industries

| Gender typed Industries | Total | | Men | | Women | |
| --- | --- | --- | --- | --- | --- | --- |
|  | aPR (95% CI) | P- Value | aPR (95% CI) | P-Value | aPR (95% CI) | P-Value |
| Female-Dominated | Ref |  | Ref |  | Ref |  |
| Gender-mixed | 1.04 (0.93-1.16) | 0.50 | 1.00 (0.84-1.19) | 0.97 | 1.04 (0.90-1.20) | 0.57 |
| Male-dominated | 0.94 (0.82-1.08) | 0.37 | 0.84 (0.69-1.02) | 0.07 | 1.09 (0.86-1.38) | 0.48 |

*Adjusted for age, civil status, and education*

*aPR = Adjusted Prevalence Ratio*
